# Electronic cigarette use among adolescents: a cross-sectional study in Hong Kong

**DOI:** 10.1186/s12889-016-2719-4

**Published:** 2016-03-01

**Authors:** Nan Jiang, Man Ping Wang, Sai Yin Ho, Lok Tung Leung, Tai Hing Lam

**Affiliations:** School of Public Health, The University of Hong Kong, 5/F William MW Mong Block, 21 Sassoon Road, Pokfulam, Hong Kong; School of Nursing, The University of Hong Kong, 4/F William MW Mong Block, 21 Sassoon Road, Pokfulam, Hong Kong

**Keywords:** Electronic cigarettes, E-cigarettes, Smoking, Adolescents, Students, Hong Kong

## Abstract

**Background:**

Little is known about electronic cigarette (e-cigarette) use among Chinese adolescents. We examined the prevalence of current (past 30-day) e-cigarette use and its associated factors in a large sample of adolescents in Hong Kong.

**Methods:**

We analyzed data of the School-based Survey on Smoking among Students 2012/13 from a representative sample of 45,857 secondary school students (mean age: 14.8 ± 1.9). We conducted chi-square tests and *t*-test to compare current e-cigarette use by covariates. We used multivariable logistic regression to examine the association between current e-cigarette use and demographic variables, parental smoking, peer smoking, knowledge about the harm of cigarette smoking, attitudes toward cigarette smoking, cigarette smoking status, use of other tobacco products, and alcohol consumption.

**Results:**

Overall, 1.1 % of students reported current e-cigarette use. Of e-cigarette users, 11.7 % were never-cigarette smokers, 15.8 % were experimental cigarette smokers, 39.3 % were former cigarette smokers, and 33.2 % were current cigarette smokers. Current e-cigarette use was associated with male sex, poor knowledge about the harm of smoking, cigarette smoking, use of other tobacco products, and alcohol consumption.

**Conclusions:**

Surveillance and intervention efforts should address a wide range of tobacco products, including e-cigarettes. Tobacco cessation programs should also address alcohol use collectively. Policies prohibiting e-cigarette sales to minors may help prevent e-cigarette uptake among adolescents.

## Background

Electronic cigarettes (e-cigarettes) are battery-powered devices that deliver aerosol (typically contain nicotine) to users by heating the cartridges which contain varying amount of nicotine, glycerol or propylene glycol, and flavouring [1]. Since their introduction to the market in the early 2000’s, e-cigarette use has increased rapidly among adolescents worldwide [2]. From 2011 to 2014, current (past 30-day) e-cigarette use has increased among US middle (0.6 % to 3.9 %) and high school (1.5 % to 13.4 %) students [3, 4], and e-cigarette has replaced cigarettes as the most popular tobacco product [4]. In Poland, ever e-cigarette use has increased among high school students from 16.8 % in 2010/11 to 62.1 % in 2013/14, and current e-cigarette use has increased from 5.5 % to 29.9 % [5]. In New Zealand, ever e-cigarette use has tripled among school students aged 14–15 years (7.0 % to 20.0 %) from 2012 to 2014 [6]. In Korea, 9.4 % of secondary school students (grade 7–12) reported ever use of e-cigarettes in 2011, and 4.7 % used e-cigarettes in the past 30 days [7]. A great proportion (72.4-76.6 %) of current e-cigarette users are dual users who also smoke conventional cigarettes, as reported by previous studies of adolescents in the US, Poland and Korea [5, 7, 8].

E-cigarettes are often marketed as a healthier alternative to cigarette smoking, smoking cessation aids, and a way to circumvent smokefree laws [9]. Smokers are motivated to use e-cigarettes because they believe that e-cigarettes are less harmful than traditional cigarettes and can help quit smoking [10–13]. However, studies on the safety and long-term health effect of e-cigarettes are limited, and research on cessation efficacy is inconsistent [2]. Most adult studies concluded that e-cigarette use was unrelated to quitting [11, 14–18] or was associated with lower odds of quitting [19, 20], whereas some claimed that e-cigarettes helped people quit [21–23]. Dutra and Glantz found that e-cigarette use was negatively associated with smoking cessation among adolescents [24]. Some public health advocates are concerned that e-cigarettes may impede the efforts of tobacco control because, first, the product may appeal to adolescents and young adults and act as a gateway to smoking due to the flavor options and stylish design. Second, it undermines clean air policies if used in smokefree environment. Third, it may re-normalize tobacco use and change social norms about smoking. Last, it may delay or prevent smokers from cessation by maintaining nicotine addiction during temporary abstinence period and deterring the use of proven-effective cessation treatments.

The first e-cigarette was invented in 2003 by a Chinese company known as Golden Dragon Group [25]. China produces 90 % of e-cigarettes worldwide [26], but the Chinese government has no regulation on e-cigarettes [27]. Hong Kong, a special administration region of China, is the most westernized city in China and has a low cigarette smoking rate. The prevalence of current smoking is 11.8 % among residents aged 15 years and above, and 3.3 % among secondary school students [28]. E-cigarettes have been available on the marketplace in Hong Kong since 2008 [29], and the retailers offer a variety of flavors (e.g., fruit, mint, chocolate, vanilla, coffee, soft drinks, tobacco, etc.) for consumers to choose from [30]. E-cigarette use is prohibited in statutory smokefree venues (e.g., indoor areas of restaurants and bars) in Hong Kong [31]. Nicotine-containing e-cigarettes are regarded as pharmaceutical products, which must be registered before sale [32]. As of December 2015, no one has applied for a license to sell nicotine-containing e-cigarettes. However, people can easily get nicotine-containing e-cigarettes through Internet such as taobao.com, a large online shopping destination in China. Nicotine-free (claimed by sellers) e-cigarettes are widely available in shopping malls and local stores [33]. Currently there is no law prohibiting e-cigarette sale to minors in Hong Kong, but the government is considering a total ban on the device, including sales, advertising, distribution, sponsorship and manufacturing [33].

Cigarette smoking is an important factor associated with e-cigarette use among young people [6, 7, 34–41]. Other correlates include male sex [6, 7, 34–40, 42], parental smoking [35, 42], peer smoking [6, 35, 42], use of alternative tobacco products [6, 37, 41] and alcohol consumption [6, 38, 41]. Given the strong relationship between cigarette smoking and e-cigarette use, psychosocial factors associated with cigarette smoking such as knowledge about smoking and attitude toward smoking [43, 44] may be also correlated with e-cigarette use, but have not been examined. Moreover, no study has examined the correlates of e-cigarette use among adolescents in Hong Kong, a region with low cigarette smoking rate. This study aimed to examine the factors associated with current e-cigarette use in a large sample of secondary school students in Hong Kong. Findings of the study will help understand the characteristics of youth e-cigarette users and inform the development of targeted interventions.

## Methods

### Data source

We analysed the data from the School-based Survey on Smoking among Students 2012/13 (hereafter referred to as “the Survey”), a biennial cross-sectional population-based sample of secondary school students (grade 7–12) in Hong Kong [28]. The Survey was approved by the Institutional Review Board of the University of Hong Kong/Hospital Authority Hong Kong West Cluster, and was carried out in accordance with the approved guidelines. The core questionnaire items were developed based on the Chinese version of Global Youth Tobacco Survey. Special schools for adolescents with visual, hearing or other physical impairments or disabilities were not included in the Survey. The Survey was commissioned by the Food and Health Bureau of the Government of the Hong Kong Special Administrative Region, and the 2012/13 data were collected from October 2012 to April 2013. The Survey was printed in Chinese (traditional characters) for local schools, and in English for international schools. The Survey employed passive parental consent procedure. An invitation letter was sent to parents of all students in the participating schools. Declining parents asked their child to return a blank questionnaire during the Survey. Student participation remained voluntary even with parental consent.

The Survey used proportionate stratified sampling to obtain a representative sample of secondary schools in all 18 districts of Hong Kong, and all students in participating schools were invited to complete an anonymous self-administered questionnaire. The sample included 45,857 students from 75 secondary schools. The response rate was 19 % at school level and 96 % at student level. School refusals were mainly related to administrative reasons. We calculated Cohen’s effect size to compare the characteristics (i.e., age, sex and grade) of our sample with official 2012/13 student enrollment data reported by the Hong Kong Education Bureau. The effect size ranged from 0.05 to 0.21, indicating that our sample was representative of the secondary school student population in Hong Kong [45].

### Main measures

The dependent variable was current e-cigarette use which was measured by one question, “In the past 30 days, which of the following products have you used: cigarettes; e-cigarettes; waterpipe; chewing tobacco; cigars; snus; smoking pipe or snuff; other tobacco product?” Students who checked e-cigarettes were defined as current e-cigarette users [46].

Independent variables included students’ age, sex, parental smoking, peer smoking, knowledge about the harm of cigarette smoking, attitudes toward cigarette smoking, cigarette smoking, other tobacco use, and alcohol consumption. Parental smoking was dichotomized as “yes” (at least one smoking parent) and “no”. Peer smoking was measured by asking, “How many of your close friends smoke?” with 5 response options from “none” to “all”. Responses were re-categorized into 3 groups (i.e., none, less than half, half or more).

Knowledge about the harm of smoking was measured by a question “Do you think smoking is harmful?” with 4 response options from “definitely no” to “definitely yes”. We dichotomized the responses as “good” if students checked “definitely yes”, and “poor” if other options were checked. Students reported their attitudes toward cigarette smoking on a 5-point Likert scale ranging from “very negative” to “very positive”. Responses were re-classified as neutral, negative, and positive.

Cigarette smoking was measured by asking, “Which statement best describes your status?” to which students could response “I have never smoked”, “I smoked once or a few times (for fun or to try a puff)”, “I used to smoke (not every day), but have quit now”, “I used to smoke every day, but have quit now”, “I smoke on some days now”, and “I smoke every day now”. Students also reported the number of smoking days within the past 30 days. We categorized the respondents into 4 groups, including never smoker, experimental smoker (smoked once or a few times), former smoker (smoked in the past but not now), and current smoker (smoke now and ≥1 smoking day in the past 30 days).

Other tobacco product use included waterpipe, chewing tobacco, cigars, snus, smoking pipe or snuff, and any other forms of tobacco (other than cigarettes and e-cigarettes). Alcohol use was measured by a question, “How frequently do you drink alcohol or alcoholic beverage?” with 6 response options from “I do not drink” to “I drink every day”. We re-categorized the responses into 3 groups, including non-drinkers, <1 day/month, and ≥1 day/month.

### Statistical analysis

Data were weighted using Hong Kong Education Bureau’s student enrolment statistics for 2012/13 to adjust for nonresponse and to match sample characteristics (i.e., age, sex, and grade) with the secondary school student population in Hong Kong. We summarized the characteristics of the sample, e-cigarette users and nonusers using descriptive statistics. We conducted chi-square tests and *t*-test to compare e-cigarette use by covariates, and conducted multivariable logistic regression to examine the association between current e-cigarette use and demographic factors, parental smoking, peer smoking, smoking knowledge and attitudes, cigarette smoking, use of other tobacco products, and alcohol consumption. Stata version 13.1 was used for data analysis.

## Results

With a mean age of 14.8 years (SD = 1.9), our sample was about equally split by sex with 48.6 % girls (Table 1). One-third (31.3 %) of the students reported that at least one of their parents smoked, and 39.1 % had close friends who smoked cigarettes. Most students (89.1 %) responded “definitely yes” to the question about the harm of cigarette smoking, and 75.8 % reported negative (or very negative) attitudes toward cigarette smoking. Most (85.1 %) of students had never smoked a  cigarette, 7.6 % were experimental smokers, 3.9 % were former smokers, and 3.3 % were current smokers. Overall, 3.0 % of the students used other tobacco products (other than cigarettes and e-cigarettes) in the past 30 days, and 33.2 % were alcohol drinkers.

**Table 1:** Characteristics of students, current e-cigarette users and nonusers in Hong Kong

	Full sample		Current e-cigarette users^a^		Nonusers	
	(N = 45857; 100 %)		(n = 560; 1.1 %)		(n = 45297; 98.9 %)	
	No.	(%)	No.	(%)	No.	(%)
Age, mean (SD)	14.8	(1.9)	15.4	(2.0)	14.8	(1.9)
Sex						
Girl	21117	(48.6)	166	(29.3)	20951	(48.8)
Boy	24740	(51.4)	394	(70.7)	24346	(51.2)
Parental smoking						
No	30648	(68.7)	315	(60.2)	30333	(68.8)
Yes	15209	(31.3)	245	(39.8)	14964	(31.2)
Peer smoking						
No	27149	(60.9)	106	(18.3)	27043	(61.4)
Less than half	14568	(30.8)	195	(36.4)	14373	(30.8)
Half or more	4068	(8.3)	254	(45.3)	3814	(7.9)
Knowledge about the harm of cigarette smoking						
Good	40604	(89.1)	312	(56.1)	40292	(89.5)
Poor	5235	(10.9)	248	(43.9)	4987	(10.5)
Attitudes toward smoking						
Negative	33878	(75.8)	167	(31.7)	33711	(76.3)
Neutral	10454	(21.4)	248	(44.1)	10206	(21.1)
Positive	1304	(2.8)	140	(24.2)	1164	(2.5)
Cigarette smoking^b^						
Never smoker	37740	(85.1)	65	(11.7)	37675	(85.9)
Experimental smoker	3721	(7.6)	77	(15.8)	3644	(7.5)
Former smoker	1899	(3.9)	153	(39.3)	1746	(3.6)
Current smoker	1694	(3.3)	171	(33.2)	1523	(3.0)
Other tobacco use^c^						
No	44459	(97.0)	323	(57.6)	44136	(97.4)
Yes	1398	(3.0)	237	(42.4)	1161	(2.6)
Alcohol consumption						
Non-drinker	29633	(66.8)	103	(16.0)	29530	(67.4)
<1 day/month	8221	(18.4)	81	(15.9)	8140	(18.4)
≥1 day/month	6846	(14.8)	354	(68.1)	6492	(14.2)

*Note*

^a^Current e-cigarette users reported e-cigarette use within the past 30 days

^b^Never smokers have never smoked a cigarette; Experimental smokers smoked once or a few times; Former smokers smoked in the past but not now; Current smokers reported smoking now and smoked on at least one day in the past 30 days

^c^Other tobacco use includes using any of the following in the past 30 days: waterpipe, chewing tobacco, cigars, snus, smoking pipe/snuff, or any other form of tobacco (other than cigarettes and e-cigarettes)

Overall, 1.1 % (95 % confidence interval [CI]: 1.0-1.3) of students used e-cigarettes during the past 30 days, and e-cigarette was the most popular alternative tobacco product (non-cigarette) used by students after waterpipe (1.2 %; data not shown in tables). Of current e-cigarettes users, 39.3 % were former cigarette smokers, 33.2 % were dual users who also smoked conventional cigarettes, 15.8 % were experimental cigarette smokers, and 11.7 % were never cigarette smokers. Of former or current cigarette smokers, 9.6 % reported current e-cigarette use (Table 2). E-cigarette users were older than nonusers (15.4 vs. 14.8 years, *p* < .001), and e-cigarette use was more common in boys (*p* < .001), students who had smoking parents (*p* = .003) or smoking peers (*p* < .001), students with poor knowledge about the harm of smoking (*p* < .001) or positive attitudes toward smoking (*p* < .001), former or current cigarette smokers (*p* < .001), other tobacco product users (*p* < .001), and alcohol users (including both students who drank <1 day/month and those who drank ≥1 day/month; *p* < .001).

**Table 2:** Prevalence of current e-cigarette use and its associated factors among adolescents in Hong Kong

	%	(95 % CI)	*P-*value for *x* ^2^ or *t*-test	AOR^a^ (95 % CI)
Age, Mean (SD)	15.4	(2.0)	<.001	0.96 (0.88, 1.05)
Sex			<.001	
Girl	0.7	(0.5, 0.9)		1
Boy	1.6	(1.4, 1.8)		1.94 (1.38, 2.73)^**^
Parental smoking			.003	
No	1.0	(0.8, 1.2)		1
Yes	1.4	(1.2, 1.7)		1.00 (0.73, 1.37)
Peer smoking			<.001	
No	0.3	(0.2, 0.4)		1
Less than half	1.3	(1.1, 1.6)		1.16 (0.69, 1.96)
Half or more	6.1	(5.1, 7.3)		1.28 (0.72, 2.27)
Knowledge about the harm of cigarette smoking			<.001	
Good	0.7	(0.6, 0.8)		1
Poor	4.6	(3.8, 5.5)		1.67 (1.16, 2.40)^*^
Attitudes toward smoking			<.001	
Negative	0.5	(0.4, 0.6)		1
Neutral	2.3	(1.9, 2.8)		0.98 (0.66, 1.45)
Positive	9.9	(7.7, 12.5)		1.09 (0.62, 1.92)
Cigarette smoking^b^			<.001	
Never smoker	0.1	(0.1, 0.2)		1
Experimental smoker	2.0	(1.5, 2.7)		9.22 (5.57, 15.27)^**^
Former smoker	9.6	(7.5, 12.1)		23.33 (13.38, 40.68)^**^
Current smoker	9.6	(7.8, 11.9)		18.17 (10.08, 32.76)^**^
Other tobacco use^c^			<.001	
No	0.7	(0.6, 0.8)		1
Yes	15.9	(13.2, 19.0)		3.59 (2.25, 5.72)^**^
Alcohol consumption			<.001	
Non-drinker	0.3	(0.2, 0.3)		1
<1 day/month	1.0	(0.7, 1.3)		1.93 (1.26, 2.96)^*^
≥1 day/month	5.2	(4.4, 6.0)		3.16 (2.12, 4.72)^**^

*Note. AOR* adjusted odds ratio, *CI* confidence interval

^a^Multivariable logistic regression model adjusted for all variables listed in the table

^b^Never smokers have never smoked a cigarette ; Experimental smokers smoked once or a few times; Former smokers smoked in the past but not now; Current smokers reported smoking now and smoked on at least one day in the past 30 days

^c^Other tobacco use includes using any of the following in the past 30 days: waterpipe, chewing tobacco, cigars, snus, smoking pipe/snuff, or any other form of tobacco (other than cigarettes and e-cigarettes)

^*^ *p* < .01; ^**^ *p* < .001

Multivariable logistic regression showed that current e-cigarette use was associated with male sex (adjusted odds ratio [AOR] = 1.94, 95 % CI: 1.38-2.73), poor knowledge about the harm of cigarette smoking (AOR = 1.67, 95 % CI: 1.16-2.40), use of other tobacco products (AOR = 3.59, 95 % CI; 2.25-5.72), alcohol consumption (AOR = 1.93, 95 % CI: 1.26-2.96 for <1 day/month; AOR = 3.16, 95 % CI: 2.12-4.72 for ≥1 day/month), and cigarette smoking. Compared with never smokers, experimenters (AOR = 9.22, 95 % CI: 5.57-15.27), former cigarette smokers (AOR = 23.33, 95 % CI: 13.38-40.68) and current cigarette smokers (AOR = 18.17, 95 % CI: 10.08-32.76) were more likely to report current e-cigarette use.

## Discussion

This is the first study to examine the factors associated with e-cigarette use in a representative sample of adolescents in Hong Kong. The prevalence of current e-cigarette use was low. However, given the low prevalence of cigarette smoking in Hong Kong [28], the e-cigarette smoking rate of 1.1 % was alarming. It should be of concern that e-cigarette use may increase rapidly among young people, as reported in previous studies [3–5]. The government must continuously monitor the use of this emerging device.

Two-fifths (39.3 %) of current e-cigarette users were former cigarette smokers. One out of every ten former cigarette smokers reported e-cigarette use during the past month. As limited by the survey questions, we don’t know whether these former cigarette smokers used e-cigarettes to help quit cigarette smoking or initiated e-cigarette use after quitting cigarettes. But, in a recent study of Hong Kong adolescents, Wang et al. [47] found that current e-cigarette use was not associated with past-year quit attempt or quit intention. Similarly, a study of New Zealand school students found that e-cigarette use was not related to quit attempt or quit intention [6]. Thus former cigarette smokers might use e-cigarettes for purposes other than cessation-related needs. It is concerning that e-cigarette use among former cigarette smokers may lead to re-initiation of cigarette smoking and the use of other tobacco products.

One-third (33.2 %) of e-cigarette users reported concurrent use of traditional cigarettes. It is unclear whether students used nicotine-containing or nicotine-free e-cigarettes. But e-cigarettes labeled as nicotine-free may contain nicotine [48]. Nicotine is highly addictive, and the fatal human dose is 50–60 mg for adults and much lower for children [49]. Increased nicotine intake associated with dual use may potentially expose students to poisoning risks [49]. In Hong Kong, e-cigarettes in the marketplace are claimed to be nicotine-free by retailers. But no organization is responsible for inspecting the ingredients and nicotine concentration, and there is no minimum legal age for e-cigarette purchase. These loopholes should be addressed by the government.

Over one quarter (27.5 %) of current e-cigarette users were never cigarette smokers (11.7 %) or experimenters (15.8 %). This finding raises our concern that e-cigarette use among non-cigarette smokers may increase. From 2011 to 2013, the number of US middle and high school students who never smoked cigarettes but had ever used e-cigarettes has tripled from 79,000 to over 263,000 [50]. In 2010/11, 0.6 % of Polish high school students reported current e-cigarette use but never cigarette smoking , and the rate increased to 2.0 % in 2013/14 [51]. E-cigarette use among never smokers and experimenters may serve as a gateway for subsequent cigarette smoking and lead to nicotine dependence [52]. A study of secondary school students in Hong Kong found that never smokers and experimenters who used e-cigarettes were more likely to report cigarette smoking susceptibility [47]. Studies of never smoking children in the UK and US have concluded that e-cigarette use was associated intention to smoke [42, 50, 53]. A study of secondary school students in Connecticut found that many never smokers initiated with nicotine-free e-cigarettes but switched to nicotine-containing e-cigarettes later, and never smokers were more likely to switch from nicotine-free to nicotine-containing e-cigarettes than current and ever cigarette smokers, indicating that e-cigarettes among non-cigarette smokers may lead to nicotine addiction [36]. Future research is warranted to investigate how e-cigarette use affects the initiation and progression of cigarette smoking among Asian adolescents.

E-cigarette was the second most common form of alternative tobacco product (non-cigarette) used by students. We do not know what motivated our adolescents to use e-cigarettes. Previous studies have found that curiosity is the top reason for youth e-cigarette use [6, 10, 38]. Another reason is related to the perception that e-cigarettes are less harmful than cigarettes [6, 10]. Future study needs to better understand the reasons for e-cigarette use, the usage patterns, e-cigarette marketing strategies, and knowledge and perceptions about e-cigarettes among adolescents in Hong Kong.

Consistent with prior studies [6, 7, 24, 34–42], we found that current e-cigarette use was associated with cigarette smoking and male sex. Other correlates included the use of other tobacco products and alcohol consumption. As substance (e.g., tobacco, alcohol and drug) use tend to cluster in adolescents [54–56], youth who used other form(s) of tobacco products and drank alcohol might be more willing to try emerging tobacco products such as e-cigarettes. Future tobacco control efforts for adolescents should address alcohol consumption and the use of multiple tobacco products rather than focusing on cigarette smoking only.

E-cigarette smoking was also associated with poor knowledge about the harm of cigarette smoking. In Hong Kong, the school-based anti-tobacco education program focuses on preventing cigarette smoking only. The program is implemented in kindergartens, primary and secondary schools and has been an important element of the comprehensive tobacco control program since 1980s [57]. Although the current school program does not include any information about alternative tobacco products, the message regarding the negative health effects of cigarette smoking appears to be able to protect schoolchildren from using other tobacco products including e-cigarettes. As e-cigarettes are a new product, school-based programs usually do not target e-cigarettes. In a study of urban public high school students in North Carolina, 90 % of the students reported that they did not receive information about e-cigarettes in their schools [37]. Similarly, the lack of formal school-based education about e-cigarettes was also reported among adolescents in California [58]. Future school education programs must address a wide range of tobacco products, and enhance students’ knowledge about the harmfulness and addictiveness of new emerging tobacco products.

There are several limitations to this study. First, the cross-sectional study design did not allow causality inference on the relationship between e-cigarette use and independent variables. Second, the study was based on self-reported data and thus was subject to reporting errors. Third, the response rate was low at school level (19 %). However, the Cohen’s effect size indicated that the results were unlikely to be biased as school refusals were mainly due to administrative factors. Fourth, data were collected among school students and special schools (for adolescents with visual, hearing or other physical impairments or disabilities) were excluded from the Survey. Therefore findings may not be generalized to all adolescents in Hong Kong. Fifth, the study was limited by the lack of information on reasons for e-cigarette use, the usage patterns, and nicotine concentration level.

## Conclusions

This study has important implications for tobacco control interventions and research. First, despite the low prevalence of e-cigarette use, e-cigarette is the second most popular alternative tobacco product (non-cigarette) used by adolescents. The Hong Kong government should continuously monitor the pattern of e-cigarette use. Future research is warranted to examine the characteristics of e-cigarette use, the marketing tactics, and how e-cigarette use might influence the patterns of cigarette smoking and other tobacco use. Second, this study identifies the factors associated with e-cigarette use among adolescents in Hong Kong, including knowledge about the harm of cigarette smoking, cigarette smoking, use of alcohol and other tobacco products. Tobacco interventions and school programs must target the full array of tobacco products and not just cigarettes, and denormalize the use of all tobacco products. Alcohol use should be addressed collectively through tobacco cessation programs. Third, a quarter of current e-cigarette users were never cigarette smokers or experimenters, suggesting that e-cigarettes appeal to non-cigarette smokers. Policies prohibiting e-cigarette sales (including nicotine-free e-cigarettes) to minors may help prevent the uptake in adolescents.
